# Association of Prepregnancy Body Mass Index With Risk of Severe Maternal Morbidity and Mortality Among Medicaid Beneficiaries

**DOI:** 10.1001/jamanetworkopen.2022.18986

**Published:** 2022-06-28

**Authors:** Heather A. Frey, Robert Ashmead, Alyssa Farmer, Yoshie H. Kim, Cynthia Shellhaas, Reena Oza-Frank, Rebecca D. Jackson, Maged M. Costantine, Courtney D. Lynch

**Affiliations:** 1 Department of Obstetrics and Gynecology, The Ohio State University College of Medicine, Columbus; 2 Ohio Colleges of Medicine Government Resource Center, The Ohio State University, Columbus; 3 Bureau of Maternal, Child and Family Health, Ohio Department of Health, Columbus; 4 Department of Internal Medicine/Endocrinology, and Diabetes and Metabolism, The Ohio State University, Columbus

## Abstract

**Importance:**

The association between body mass index (BMI, which is calculated as weight in kilograms divided by height in meters squared) and severe maternal morbidity (SMM) and/or mortality is uncertain, judging from the current evidence.

**Objective:**

To examine the association between prepregnancy BMI and SMM and/or mortality through 1 year post partum and to identify both the direct and indirect implications of maternal obesity for SMM and/or mortality by examining hypertensive disorders and pregestational diabetes as potential mediators.

**Design, Setting, and Participants:**

This population-based cohort study was conducted from March to October 2021 using the vital records and linked Medicaid claims data in the state of Ohio from January 1, 2012, through December 31, 2017. The cohort comprised pregnant Medicaid beneficiaries who delivered at 20 weeks’ gestation or later and had prepregnancy BMI information.

**Exposures:**

The primary exposure was maternal prepregnancy BMI, which was categorized as follows: underweight (<18.5), healthy weight (18.5-24.9), overweight (25.0-29.9), class 1 obesity (30.0-34.9), class 2 obesity (35.0-39.9), and class 3 obesity (≥40.0).

**Main Outcomes and Measures:**

The primary outcome was a composite of SMM (defined using Centers for Disease Control and Prevention criteria) and/or maternal mortality between 20 weeks’ gestation and 1 year post partum. Additional periods were assessed, including 20 weeks’ gestation through delivery hospitalization and 20 weeks’ gestation through 42 days post partum. Generalized estimating equation models were used to estimate adjusted relative risks (aRRs) for the primary outcome according to BMI category. Maternal hypertensive diseases and pregestational diabetes were assessed as potential meditators.

**Results:**

In a cohort of 347 497 pregnancies among 276 691 Medicaid beneficiaries (median [IQR] maternal age at delivery, 25 [21-29] years; 210 470 non-Hispanic White individuals [60.6%]), the prevalence of maternal obesity was 30.5% (n = 106 031). Composite SMM and/or mortality outcome occurred in 5.3% of pregnancies (n = 18 398). Overweight (aRR, 1.07; 95% CI, 1.03-1.11) and obesity (class 1: aRR, 1.19 [95% CI, 1.14-1.24]; class 2: aRR, 1.37 [95% CI, 1.30-1.44]; class 3: aRR, 1.71 [95% CI, 1.63-1.80]) were associated with an elevated risk of SMM and/or mortality during pregnancy to 1 year post partum compared with healthy BMI. Similar findings were observed when the follow-up period was shortened to 42 days post partum or the delivery hospitalization. Hypertension mediated 65.1% (95% CI, 64.6%-65.6%) of the association between obesity and the primary outcome.

**Conclusions and Relevance:**

Results of this study showed that maternal prepregnancy obesity was associated with an elevated risk of SMM and/or mortality. Hypertensive disorders appeared to mediate this association, suggesting that improved prevention and management of hypertensive disorders in pregnancy may reduce morbidity and mortality in individuals with obesity.

## Key Points

**Question:**

Is there an association between prepregnancy body mass index and risk of severe maternal morbidity and/or mortality?

**Findings:**

In this cohort study of 347 497 pregnancies, individuals with prepregnancy obesity had increased risk of severe maternal morbidity and/or mortality from 20 weeks’ gestation through 1 year post partum compared with those with healthy weight. A large proportion of this association was mediated by hypertensive disorders.

**Meaning:**

Findings of this study suggest that prevention and management of hypertensive disorders in pregnancy may reduce morbidity and mortality in individuals with obesity.

## Teaser

This cohort study analyzes the role of obesity in pregnancy-related medical conditions and outcomes during the antepartum, intrapartum, and postpartum periods.

## Introduction

Obesity, defined as a body mass index (BMI, which is calculated as weight in kilograms divided by height in meters squared) of 30 or higher, is a leading obstetric comorbidity affecting 29% of pregnant individuals.^1^ Those with obesity have a greater risk of pregnancy complications, such as gestational diabetes,^2,3,4^ preeclampsia,^4,5^ postpartum hemorrhage,^6,7^ venous thromboembolism,^8,9^ and infection^10,11^ compared with those with a healthy BMI (18.5-24.9). Despite these risks, an association has not been consistently demonstrated between obesity and mortality as well as the more common near-miss cases of severe maternal morbidity (SMM) during pregnancy and the postpartum period.^12,13,14,15,16,17,18,19,20^

Contradictory findings in studies of obesity and SMM may be attributable to the variation in the definition of SMM. Yet another major limitation of previous studies is the lack of assessment of SMM and mortality beyond the delivery hospitalization. The World Health Organization defines maternal mortality as death during pregnancy or within 42 days of delivery,^21^ and the Centers for Disease Control and Prevention has expanded this definition to include deaths occurring within 365 days of delivery.^22^ It is estimated that 35% to 40% of all pregnancy-related deaths in the US occur between 7 days and 1 year post partum.^23,24^ Relative to mortality in the postpartum period, it is estimated that many more individuals experience SMM, but data on SMM beyond the delivery hospitalization are limited.^25^

In this cohort study, we comprehensively assessed the complex association between maternal BMI and SMM and/or mortality. Although obesity may be a central and common risk factor underlying multiple conditions associated with a predisposition to SMM and mortality, it is unlikely to be the direct cause of maternal death in most cases. Thus, our objectives were (1) to estimate the association between prepregnancy BMI and SMM and/or mortality through 1 year post partum and (2) to identify both the direct and indirect implications of maternal obesity for SMM and/or mortality by examining hypertensive disorders and pregestational diabetes as potential mediators. We focused on these 2 conditions because they are more prevalent among individuals with obesity and are associated with higher rates of maternal complications. We hypothesized that increased prepregnancy BMI was associated with higher SMM and/or mortality and was mediated by both hypertension and diabetes.

## Methods

### Study Design

We performed this population-based, retrospective cohort study in March to October 2021. The study was approved by the institutional review boards at The Ohio State University and the Ohio Department of Health as well as by the Data Use Committee at the Ohio Department of Medicaid, which waived the informed consent requirement because the research involved no more than minimal risk and could not practicably be carried out without the waiver of informed consent. We followed the Strengthening the Reporting of Observational Studies in Epidemiology (STROBE) reporting guideline.

The cohort consisted of all pregnancies among Medicaid beneficiaries in Ohio that resulted in a live birth or fetal death at 20 weeks’ gestation or later from January 1, 2012, through December 31, 2017. This date range was selected because, at the time of analysis, it was the most recent date for which data were available from the Ohio Department of Health Pregnancy-Associated Mortality Review (PAMR) program. To ensure that the entire risk period was observed, we excluded pregnancies of 20 weeks’ gestation or later before January 1, 2012. Pregnancies with missing maternal prepregnancy BMI information were also excluded.

### Data Linkage and Exposure

We used an existing database of Ohio live births (2012-2017) and fetal deaths (2012-2015) that had been previously linked to Medicaid claims data.^26^ To complete the study cohort, we linked fetal death data from 2016 and 2017 to the Medicaid claims data within the same period using the Link Plus Beta, version 3.0 (Centers for Disease Control and Prevention) (eAppendix in the Supplement). The unit of analysis was the pregnancy.

Maternal BMI was calculated using prepregnancy height and weight recorded in the Ohio Bureau of Vital Statistics birth and fetal death files.^27^ The primary exposure of the study was maternal prepregnancy BMI, which was categorized as follows: underweight (<18.5), healthy weight (18.5-24.9), overweight (25.0-29.9), class 1 obesity (30.0-34.9), class 2 obesity (35.0-39.9), and class 3 obesity (≥40.0).^28^

### Outcome Measures

The primary outcome of the study was a composite of SMM and/or maternal mortality between 20 weeks’ gestation and 1 year post partum. Severe maternal morbidity was defined as the presence of 1 or more of 21 indicators (eTable 1 in the Supplement) with associated *International Classification of Diseases, Ninth Revision *(*ICD-9*) and *International Statistical Classification of Diseases and Related Health Problems, Tenth Revision *(*ICD-10*) diagnosis and procedure codes that have been validated and endorsed by the Centers for Disease Control and Prevention.^22^ Given the concerns regarding the specificity of the procedure codes for blood transfusion as a marker of hemorrhage consistent with SMM,^29^ we performed a sensitivity analysis excluding SMM cases that involved only a blood transfusion.

Pregnancy-associated deaths were identified using data from the PAMR program, which uses a rigorous, multidisciplinary committee to review all cases of maternal death to ascertain a cause.^30^ In addition, each death is classified by the PAMR committee as pregnancy related or as pregnancy associated but not pregnancy related.^31,32^ The primary outcome included all maternal deaths regardless of classification, but the association between maternal obesity (BMI ≥30) and pregnancy-related classification was also evaluated.

Severe maternal morbidity and/or maternal mortality were assessed over 2 alternative periods: (1) 20 weeks’ gestation through delivery hospitalization and (2) 20 weeks’ gestation through 42 days post partum. Secondary outcomes of the study were SMM and mortality as separate categories and each of the 21 indicators in the SMM composite.

### Covariates

Maternal demographic characteristics were obtained from the birth or fetal death records. Race and ethnicity were indicated as reported in the Ohio Bureau of Vital Statistics and included the following categories: Hispanic, non-Hispanic Black, non-Hispanic White, and other (ie, American Indian or Alaskan Native, Asian, and Native Hawaiian or Other Pacific Islander). Obstetric data, including parity, tobacco use, previous cesarean delivery, mode of delivery, trimester of prenatal care initiation, and gestational age at delivery, were also abstracted. All other data regarding maternal comorbidities and delivery complications were obtained from the Medicaid claims data using *ICD-9* and *ICD-10* diagnostic and procedure codes (eTable 2 in the Supplement).

In multifetal pregnancies, covariates from the multiple birth or fetal death records were reduced into a single pregnancy record for analysis. If there were discrepancies within the vital records, we considered binary risk factors to be present if any of the records showed the risk factor. For dates of birth, gestational age, plurality, and parity, we used the earlier data or the smaller number.

### Statistical Analysis

The incidence of the composite outcome of SMM and/or mortality through 1 year post partum per 10 000 pregnancies was calculated for each BMI category (underweight, healthy weight, overweight, class 1 obesity, class 2 obesity, and class 3 obesity). Incidence rates of each SMM indicator and mortality were reported as separate secondary outcomes. The outcomes were assessed over 2 alternative periods (through delivery hospitalization and through 42 days post partum).

Generalized estimating equation models with a log link were used to calculate adjusted relative risks (aRRs) for the primary composite outcome according to BMI category during each of the 3 periods. Multiple pregnancies of the same individual were accounted for in the model by using an AR(1) correlation structure with robust SEs. Covariates were chosen by examination of a directed acyclic graph (eFigure in the Supplement).^33^ Race and ethnicity as socially constructed variables were included in the model because individual and structural racism likely affect maternal morbidity and mortality.^34^ Missing covariates were multiply-imputed using a sequential hot-deck approach, and estimates were combined using Rubin rules^35^ with 50 imputations. Multicollinearity among factors was examined via pairwise correlations.

To further understand the association between maternal obesity and SMM and/or mortality, we used regression-based causal mediation methods^36^ to evaluate the role of maternal hypertensive diseases and pregestational diabetes in the association between maternal obesity and the primary outcome, while controlling for the same confounders as the generalized estimating equation models (eAppendix in the Supplement). In this analysis, hypertensive disease was defined as chronic hypertension, preeclampsia, and/or gestational hypertension.

The tests were 2-tailed, and *P* < .05 indicated statistical significance. All statistical analyses were performed using R, version 4.1.0 (R Foundation for Statistical Computing).^37^

## Results

### Study Population

There were 389 161 live births or fetal deaths linked to Medicaid beneficiaries in Ohio from 2012 to 2017. After excluding records with missing prepregnancy BMI data (n = 12 526), gestational age of 20 weeks or later on January 1, 2012 (n = 23 477), and delivery earlier than 20 weeks’ gestation (n = 674), and then combining the records from multifetal gestation into a single pregnancy record, we were left with a final analytic cohort of 347 497 pregnancies among 276 691 Medicaid beneficiaries (Figure). Of these beneficiaries, the median (IQR) maternal age at delivery was 25 (21-29) years, and 210 470 (60.6%) had non-Hispanic White race and ethnicity.

**Figure.**
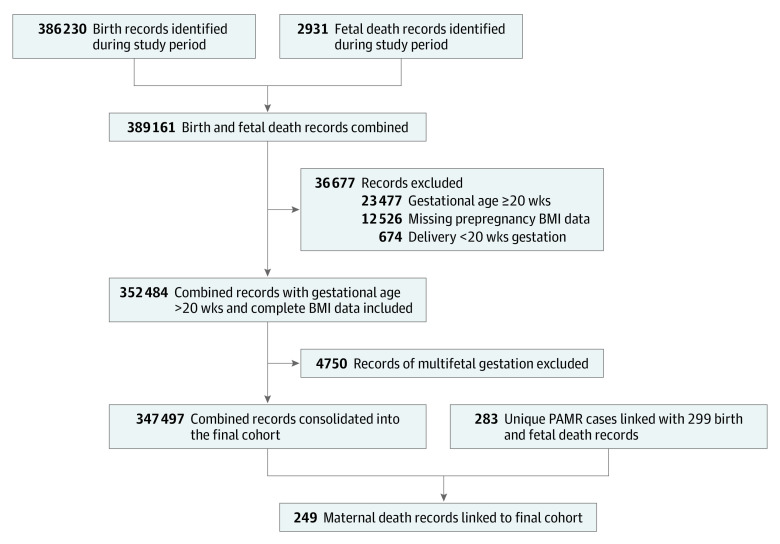
Study Flow Diagram BMI indicates body mass index; PAMR, Pregnancy-Associated Mortality Review.

The prevalence of maternal obesity was 30.5% (n = 106 031 of 347 497). Beneficiaries with obesity were more likely to be non-Hispanic Black, older, more educated, and married compared with those with healthy weight (Table 1). They also had a higher prevalence of diabetes, chronic hypertension, preeclampsia or gestational hypertension, and previous cesarean delivery. In addition, more pregnancies complicated by maternal obesity resulted in cesarean delivery. For example, there were 32 185 (23.0%) cesarean deliveries recorded among 140 132 beneficiaries with healthy weight compared with 12 452 (49.9%) cesarean deliveries among 24 973 beneficiaries with class 3 obesity. Tobacco use, illicit substance use, and nulliparity were more common among those with healthy weight. The proportion of individuals who were continuously enrolled in Medicaid through 42 days post partum did not differ by prepregnancy obesity (95.5% of those with BMI <30 compared with 96.6% of those with BMI ≥30). There was no difference by prepregnancy obesity status in the proportion of individuals who were continuously enrolled through 1 year post partum (80.0% of those with BMI <30 vs 82.7% of those with BMI ≥30).

**Table 1.** Maternal Demographic and Index Pregnancy Characteristics by Prepregnancy BMI

Characteristic	No. (%)					
	BMI <18.5 (n = 17 476)	BMI 18.5-24.9 (n = 140 132)	BMI 25.0-29.9 (n = 83 858)	BMI 30.0-34.9 (n = 52 245)	BMI 35.0-39.9 (n = 28 813)	BMI ≥40 (n = 24 973)
Maternal demographic characteristics						
Maternal age, y						
<20	3432 (19.6)	21 348 (15.2)	8705 (10.4)	4460 (8.5)	1897 (6.6)	1108 (4.4)
20-24	7383 (42.2)	54 300 (38.7)	29 718 (35.4)	18 343 (35.1)	9847 (34.2)	7642 (30.6)
25-29	4316 (24.7)	38 277 (27.3)	24 923 (29.7)	15 951 (30.5)	9250 (32.1)	8528 (34.1)
30-34	1748 (10.0)	18 339 (13.1)	13 835 (16.5)	8938 (17.1)	528518.3)	5148 (20.6)
35-39	506 (2.9)	6507 (4.6)	5467 (6.5)	3714 (7.1)	2083 (7.2)	2118 (8.5)
≥40	91 (0.5)	1360 (1.0)	1207 (1.4)	839 (1.6)	450 (1.6)	429 (1.7)
Missing data	0	1 (<0.1)	3 (<0.1)	0	1 (<0.1)	0
Race and ethnicity^a^						
Hispanic	750 (4.3)	8873 (6.3)	6604 (7.9)	3855 (7.4)	1775 (6.2)	1282 (5.1)
Non-Hispanic Black	3673 (21.0)	35 263 (25.2)	24 815 (29.6)	16 158 (30.9)	8928 (31.0)	8075 (32.3)
Non-Hispanic White	11 958 (68.4)	88 336 (63.0)	48 413 (57.7)	30 026 (57.5)	17 041 (59.1)	14 696 (58.8)
Other^b^	700 (4.0)	4073 (2.9)	1843 (2.2)	821 (1.6)	266 (0.9)	206 (0.8)
Missing data	395 (2.3)	3587 (2.6)	2183 (2.6)	1385 (2.7)	803 (2.8)	714 (2.9)
Educational level						
<High school diploma	5107 (29.2)	32 518 (23.2)	17 622 (21.0)	10 167 (19.5)	5223 (18.1)	4028 (16.1)
High school diploma	7223 (41.3)	54 999 (39.2)	32 715 (39.0)	20 700 (39.6)	11 567 (40.1)	9945 (39.8)
Some college	3752 (21.5)	35 938 (25.6)	22 728 (27.1)	14 930 (28.6)	8275 (28.7)	7683 (30.8)
≥College degree	1327 (7.6)	16 189 (11.6)	10 447 (12.5)	6251 (12.0)	3650 (12.7)	3244 (13.0)
Missing data	67 (0.4)	488 (0.3)	346 (0.4)	197 (0.4)	98 (0.3)	73 (0.3)
Marital status						
Married	3772 (21.6)	33 263 (23.7)	22 325 (26.6)	14 241 (27.3)	8358 (29.0)	7725 (30.9)
Not married	13 703 (78.4)	106 860 (76.3)	61 527 (73.4)	38 002 (72.7)	20 451 (71.0)	17 246 (69.1)
Missing data	1 (<0.1)	9 (<0.1)	6 (<0.1)	2 (<0.1)	4 (<0.1)	2 (<0.1)
County of residence						
Metropolitan	10 071 (57.6)	85 628 (61.1)	53 232 (63.5)	32 790 (62.8)	17 801 (61.8)	15 351 (61.5)
Suburban	2263 (12.9)	17 178 (12.3)	9520 (11.4)	5959 (11.4)	3214 (11.2)	2807 (11.2)
Rural	2185 (12.5)	16 616 (11.9)	9423 (11.2)	6097 (11.7)	3495 (12.1)	3107 (12.4)
Appalachian	2946 (16.9)	20 632 (14.7)	11 659 (13.9)	7382 (14.1)	4298 (14.9)	3705 (14.8)
Missing data	11 (0.1)	78 (0.1)	24 (<0.1)	17 (<0.1)	5 (<0.1)	3 (<0.1)
Pregnancy characteristics						
Prenatal care in first trimester	9891 (56.6)	81 229 (58.0)	50 820 (60.6)	32 596 (62.4)	18 794 (65.2)	16 684 (66.8)
Parity						
First pregnancy	6538 (37.4)	45 193 (32.3)	22 078 (26.3)	12 512 (23.9)	6749 (23.4)	5546 (22.2)
Previous pregnancy, no live births	1382 (7.9)	10 571 (7.5)	5789 (6.9)	3474 (6.6)	1814 (6.3)	1711 (6.9)
1 Previous live birth	4760 (27.2)	40 677 (29.0)	24 768 (29.5)	15 861 (30.4)	8844 (30.7)	7824 (31.3)
>1 Previous live birth	4796 (27.4)	43 691 (31.2)	31 223 (37.2)	20 398 (39.0)	11 406 (39.6)	9892 (39.6)
Multifetal gestation	153 (0.9)	1631 (1.2)	1171 (1.4)	807 (1.5)	503 (1.7)	485 (1.9)
Tobacco use in 3 mo before pregnancy						
No	9737 (55.7)	90 303 (64.4)	57 083 (68.1)	35 848 (68.6)	20 017 (69.5)	17 881 (71.6)
Yes	7573 (43.3)	48 488 (34.6)	26 033 (31.0)	15 900 (30.4)	8541 (29.6)	6842 (27.4)
Missing data	166 (0.9)	1341 (1.0)	742 (0.9)	497 (1.0)	255 (0.9)	250 (1.0)
Illicit substance use	2151 (12.3)	14 980 (10.7)	7115 (8.5)	3816 (7.3)	1806 (6.3)	1384 (5.5)
Diabetes						
Pregestational	240 (1.4)	2362 (1.7)	2626 (3.1)	2640 (5.1)	2167 (7.5)	2831 (11.3)
Gestational	1390 (8.0)	12567 (9.0)	11 118 (13.3)	9451 (18.1)	6376 (22.1)	6742 (27.0)
Hypertension						
Chronic	304 (1.7)	3761 (2.7)	4411 (5.3)	4529 (8.7)	3833 (13.3)	5561 (22.3)
Preeclampsia or gestational	1014 (5.8)	11 132 (7.9)	9894 (11.8)	8279 (15.8)	5892 (20.4)	6720 (26.9)
Cardiac disease	534 (3.1)	3865 (2.8)	2391 (2.9)	1636 (3.1)	1105 (3.8)	1052 (4.2)
Previous cesarean delivery						
No	15 803 (90.4)	123 697 (88.3)	70 623 (84.2)	42 224 (80.8)	22369 (77.6)	18 128 (72.6)
Yes	1586 (9.1)	15 867 (11.3)	12 966 (15.5)	9884 (18.9)	6369 (22.1)	6801 (27.2)
Missing data	87 (0.5)	568 (0.4)	269 (0.3)	137 (0.3)	75 (0.3)	44 (0.2)
Index delivery characteristics						
Preterm birth <37 wks	2539 (14.5)	15 474 (11.0)	8823 (10.5)	5621 (10.8)	3360 (11.7)	3039 (12.2)
Stillbirth	60 (0.3)	824 (0.6)	566 (0.7)	409 (0.8)	280 (1.0)	260 (1.0)
Mode of delivery						
Spontaneous vaginal	12 963 (74.2)	100 532 (71.7)	56 014 (66.8)	32 084 (61.4)	16 098 (55.9)	11 836 (47.4)
Operative vaginal	1085 (6.2)	7354 (5.2)	3521 (4.2)	1902 (3.6)	893 (3.1)	665 (2.7)
Cesarean	3422 (19.6)	32 185 (23.0)	24 289 (29.0)	18 232 (34.9)	11 810 (41.0)	12 452 (49.9)
Unknown	6 (<0.1)	61 (<0.1)	34 (<0.1)	27 (0.1)	12 (<0.1)	20 (0.1)
Year of delivery						
2012	2092 (12.0)	16 136 (11.5)	9019 (10.8)	5470 (10.5)	2948 (10.2)	2538 (10.2)
2013	3112 (17.8)	24 742 (17.7)	14 262 (17.0)	8686 (16.6)	4731 (16.4)	4135 (16.6)
2014	3148 (18.0)	25 229 (18.0)	14 746 (17.6)	9167 (17.5)	4926 (17.1)	4192 (16.8)
2015	3049 (17.4)	24 805 (17.7)	15 030 (17.9)	9322 (17.8)	5224 (18.1)	4533 (18.2)
2016	3058 (17.5)	24 475 (17.5)	14 969 (17.9)	9592 (18.4)	5332 (18.5)	4611 (18.5)
2017	3017 (17.3)	24 745 (17.7)	15 832 (18.9)	10 008 (19.2)	5652 (19.6)	4964 (19.9)

Abbreviation: BMI, body mass index (calculated as weight in kilograms divided by height in meters squared).

^a^ Race and ethnicity were indicated as reported in the Ohio Bureau of Vital Statistics.

^b^ Other category included American Indian or Alaska Native, Asian, and Native Hawaiian or Other Pacific Islander.

### Main Analyses

The composite outcome of SMM and/or mortality occurred in 5.3% of pregnancies (n = 18 398 of 347 497) from 20 weeks’ gestation through 1 year post partum. There were 18 293 pregnancies complicated by SMM (526 per 10 000 pregnancies), whereas there were 249 maternal deaths (7.2 per 10 000 pregnancies). Only 21 deaths (8.4%) occurred before or during the delivery hospitalization, 40 deaths (16.1%) occurred in the first 42 days post partum, and 188 deaths (75.5%) occurred between 42 days and 1 year post partum (eTable 3 in the Supplement). Among the maternal deaths, 22.9% (n = 57) were classified as pregnancy related, 62.2% (n = 155) were pregnancy associated but not related, and 14.9% (n = 37) could not be classified by the PAMR committee. Most deaths were caused by unintentional injury (51.8%; n = 129), and maternal drug use was a risk factor in 36.5% (n = 91) of all deaths. Deaths among beneficiaries with obesity were more likely to be classified as pregnancy related compared with deaths among those without obesity (30.4% [n = 24] vs 19.4% [n = 33]) (eTable 3 in the Supplement). The timing of maternal death relative to the delivery was similar among beneficiaries with and without obesity.

The incidence of the composite outcome of SMM and/or mortality from 20 weeks’ gestation through 1 year post partum was higher among those with obesity vs those with healthy weight (6.4% [n = 6806] vs 4.6% [n = 6512]), and it was the highest among those with class 3 obesity (8.1% [n = 2036]; 815.3 per 10 000 pregnancies; 95% CI, 781.3-849.2 per 10 000 pregnancies) (Table 2). Mortality rates were slightly higher among beneficiaries with underweight (10.3 per 10 000 pregnancies; 95% CI, 5.5-15.1 per 10 000 pregnancies) and class 3 obesity (8.8 per 10 000 pregnancies; 95% CI, 5.1-12.5 per 10 000 pregnancies) compared with a rate of 7.0 per 10 000 pregnancies (95% CI, 5.6-8.4 per 10 000 pregnancies) in those with healthy weight.

**Table 2.** Unadjusted Incidence of SMM and/or Mortality Through 1 Year Post Partum per 10 000 Pregnancies by Prepregnancy BMI

Outcome	No. (No. per 10 000 pregnancies)					
	BMI <18.5 (n = 17 476)	BMI 18.5-24.9 (n = 140 132)	BMI 25.0-29.9 (n = 83 858)	BMI 30.0-34.9 (n = 52 245)	BMI 35.0-39.9 (n = 28 813)	BMI ≥40 (n = 24 973)
Composite of SMM and/or mortality	854 (488.7)	6512 (464.7)	4226 (503.9)	2921 (559.1)	1849 (641.7)	2036 (815.3)
Composite excluding transfusion	697 (398.8)	5377 (383.7)	3614 (431.0)	2572 (492.3)	1645 (570.9)	1870 (748.8)
Mortality	18 (10.3)	98 (7.0)	54 (6.4)	39 (7.5)	18 (6.2)	22 (8.8)
AMI	16 (9.2)	106 (7.6)	65 (7.8)	46 (8.8)	36 (12.5)	38 (15.2)
Aneurysm	6 (3.4)	48 (3.4)	19 (2.3)	20 (3.8)	10 (3.5)	12 (4.8)
Acute kidney failure	69 (39.5)	536 (38.2)	344 (41.0)	252 (48.2)	147 (51.0)	154 (61.7)
ARDS	104 (59.5)	777 (55.4)	446 (53.2)	316 (60.5)	200 (69.4)	243 (97.3)
Amniotic fluid embolism	2 (1.1)	17 (1.2)	18 (2.1)	16 (3.1)	6 (2.1)	3 (1.2)
Cardiac arrest or ventricular fibrillation	23 (13.2)	137 (9.8)	76 (9.1)	45 (8.6)	29 (10.1)	30 (12.0)
Conversion of cardiac rhythm	5 (2.9)	29 (2.1)	19 (2.3)	16 (3.1)	8 (2.8)	6 (2.4)
DIC	115 (65.8)	953 (68.0)	616 (73.5)	381 (72.9)	245 (85.0)	253 (101.3)
Eclampsia	64 (36.6)	438 (31.3)	350 (41.7)	274 (52.4)	180 (62.5)	197 (78.9)
Heart failure or arrest in surgery or procedure	23 (13.2)	137 (9.8)	76 (9.1)	45 (8.6)	29 (10.1)	30 (12.0)
Cerebrovascular event	88 (50.4)	698 (49.8)	466 (55.6)	330 (63.2)	196 (68.0)	211 (84.5)
Pulmonary edema or acute heart failure	71 (40.6)	520 (37.1)	408 (48.7)	299 (57.2)	226 (78.4)	299 (119.7)
Severe anesthesia complication	3 (1.7)	27 (1.9)	20 (2.4)	15 (2.9)	5 (1.7)	13 (5.2)
Sepsis	202 (115.6)	1540 (109.9)	926 (110.4)	689 (131.9)	427 (148.2)	543 (217.4)
Shock	13 (7.4)	75 (5.4)	46 (5.5)	24 (4.6)	10 (3.5)	9 (3.6)
Sickle cell disease with crisis	60 (34.3)	343 (24.5)	176 (21.0)	127 (24.3)	78 (27.1)	68 (27.2)
Air or thrombotic embolism	55 (31.5)	495 (35.3)	421 (50.2)	261 (50.0)	204 (70.8)	279 (111.7)
Blood transfusion	205 (117.3)	1497 (106.8)	813 (96.9)	489 (93.6)	278 (96.5)	246 (98.5)
Hysterectomy	28 (16.0)	171 (12.2)	124 (14.8)	102 (19.5)	65 (22.6)	58 (23.2)
Temporary tracheostomy	7 (4.0)	16 (1.1)	8 (1.0)	3 (0.6)	2 (0.7)	3 (1.2)
Ventilation	64 (36.6)	346 (24.7)	187 (22.3)	134 (25.6)	81 (28.1)	108 (43.2)

Abbreviations: AMI, acute myocardial infarction; ARDS, acute respiratory distress syndrome; BMI, body mass index (calculated as weight in kilograms divided by height in meters squared); DIC, disseminated intravascular coagulation; SMM, severe maternal morbidity.

Overall, sepsis was the most common SMM outcome encountered, followed by blood transfusion. Rates of sepsis, heart failure or pulmonary edema, eclampsia, disseminated intravascular coagulation, and air or thrombotic embolism were highest among beneficiaries with obesity, particularly those with class 3 obesity. After adjusting for confounders, overweight (aRR, 1.07; 95% CI, 1.03-1.11) and obesity (class 1: aRR, 1.19 [95% CI, 1.14-1.24]; class 2: aRR, 1.37 [95% CI, 1.30-1.44]; class 3: aRR, 1.71 [95% CI, 1.63-1.80]) were associated with a significantly higher risk of SMM and/or mortality compared with healthy weight (Table 3).

**Table 3.** Unadjusted and Adjusted Risk of Severe Maternal Morbidity and/or Mortality by Prepregnancy BMI

Outcome	BMI <18.5		BMI 25.0-29.9		BMI 30.0-34.9		BMI 35.0-39.9		BMI ≥40	
	RR (95% CI)^a^	aRR (95% CI)^b^	RR (95% CI)^a^	aRR (95% CI)^b^	RR (95% CI)^a^	aRR (95% CI)^b^	RR (95% CI)^a^	aRR (95% CI)^b^	RR (95% CI)^a^	aRR (95% CI)^b^
Through 1 y after delivery										
Composite of SMM and/or mortality	1.05 (0.98-1.13)	1.05 (0.98-1.13)	1.08 (0.98-1.13)	1.07 (1.03-1.11)	1.20 (1.15-1.26)	1.19 (1.14-1.24)	1.38 (1.31-1.45)	1.37 (1.30-1.44)	1.75 (1.67-1.84)	1.71 (1.63-1.80)
Composite excluding transfusion	1.04 (0.96-1.12)	1.04 (0.96-1.12)	1.12 (1.08-1.17)	1.11 (1.06-1.15)	1.28 (1.23-1.34)	1.26 (1.20-1.32)	1.49 (1.41-1.57)	1.46 (1.38-1.54)	1.95 (1.86-2.05)	1.88 (1.78-1.98)
Mortality	1.47 (0.89-2.43)	1.42 (0.86-2.34)	0.92 (0.66-1.28)	0.95 (0.68-1.33)	1.07 (0.74-1.55)	1.13 (0.78-1.64)	0.89 (0.54-1.48)	0.96 (0.58-1.58)	1.26 (0.79-2.00)	1.33 (0.84-2.12)
Through 42 d after delivery										
Composite of SMM and/or mortality	1.03 (0.95-1.12)	1.03 (0.95-1.12)	1.09 (1.04-1.14)	1.07 (1.02-1.12)	1.21 (1.15-1.28)	1.19 (1.13-1.25)	1.42 (1.34-1.50)	1.39 (1.31-1.47)	1.83 (1.73-1.93)	1.76 (1.66-1.86)
Composite excluding transfusion	0.99 (0.90-1.10)	1.01 (0.91-1.11)	1.15 (1.09-1.21)	1.12 (1.06-1.18)	1.33 (1.26-1.41)	1.29 (1.22-1.37)	1.58 (1.48-1.68)	1.53 (1.43-1.63)	2.13 (2.01-2.26)	2.01 (1.89-2.14)
Through delivery hospitalization										
Composite of SMM and/or mortality	1.06 (0.96-1.16)	1.07 (0.97-1.18)	1.05 (1.00-1.11)	1.03 (0.98-1.09)	1.09 (1.02-1.16)	1.07 (1.00-1.13)	1.29 (1.21-1.39)	1.27 (1.18-1.37)	1.50 (1.40-1.61)	1.45 (1.35-1.56)
Composite excluding transfusion	1.05 (0.94-1.18)	1.07 (0.95-1.20)	1.11 (1.04-1.18)	1.08 (1.01-1.15)	1.20 (1.12-1.29)	1.16 (1.08-1.25)	1.48 (1.36-1.60)	1.43 (1.31-1.55)	1.79 (1.66-1.94)	1.70 (1.57-1.85)

Abbreviations: aRR, adjusted relative risk; BMI, body mass index (calculated as weight in kilograms divided by height in meters squared); RR, relative risk; SMM, severe maternal morbidity.

^a^ Referent group was BMI of 18.5 to 24.9.

^b^ Adjusted for maternal age, race and ethnicity, educational level, marital status, parity, tobacco use, illicit substance use, and county of residence.

A similar association between maternal BMI and SMM and/or mortality was seen in the other periods assessed (Table 3). The risk of the composite outcome remained highest among those with class 3 obesity when the period of follow-up was 42 days post partum (aRR, 1.76; 95% CI, 1.66-1.86) and delivery hospitalization (aRR, 1.45; 95% CI, 1.35-1.56). In the period from 20 weeks’ gestation through 42 days post partum, blood transfusion was the most common SMM, except in individuals with class 2 (97.5 per 10 000 pregnancies [n = 281]) or class 3 (156.2 per 10 000 pregnancies [n = 390]) obesity in whom sepsis was more frequently diagnosed (eTable 4 in the Supplement). When assessed through the delivery hospitalization only (eTable 5 in the Supplement), most cases of SMM were owing to blood transfusion, and the incidence of disseminated intravascular coagulation, eclampsia, heart failure or pulmonary edema, and air or thrombotic embolism increased with higher maternal BMI. The incidence of SMM also remained higher in individuals with obesity compared with those without obesity when evaluated in each of the nonoverlapping periods: antepartum (1.3% vs 0.9%), delivery hospitalization (2.1% vs 1.8%), hospital discharge to 42 days post partum (2.0% vs 1.1%), and 42 days to 1 year post partum (2.4% vs 1.7%) (eTable 6 in the Supplement).

### Mediation Analysis

To better understand the roles of hypertensive disorders and pregestational diabetes, we performed a regression-based mediation analysis. For SMM and/or mortality, 65.1% (95% CI, 64.6%-65.6%) of the association with maternal obesity was mediated by hypertension, whereas 9.1% (95% CI, 8.7%-9.4%) was mediated by pregestational diabetes (eTable 7 in the Supplement). For SMM and/or mortality without blood transfusion in the composite outcome measure, 58.5% (95% CI 58.0%-59.0%) of the association with maternal BMI was mediated by hypertensive disorders, whereas 8.0% (95% CI, 7.7%-8.4%) was mediated by pregestational diabetes.

## Discussion

This population-based cohort study found that maternal obesity was independently associated with SMM and/or mortality during pregnancy through 1 year post partum. The findings were similar when the postpartum follow-up period was confined to 42 days after delivery and delivery hospitalization. The risk was highest in the group of Medicaid beneficiaries with class 3 obesity, 8.1% of whom experienced SMM and/or mortality during pregnancy or in the year after delivery. Results of the mediation analysis suggested that much of the association of obesity with SMM and/or mortality that beneficiaries experienced was mediated by hypertensive complications.

Evidence of the association between maternal BMI and SMM and/or mortality has been conflicting. In a secondary analysis of more than 115 000 patients enrolled at 25 US medical centers, maternal obesity was not independently associated with SMM during the delivery hospitalization.^13^ This lack of independent association between maternal BMI and SMM and/or mortality has been reported in other large studies as well.^15,19^ In contrast, Lisonkova and colleagues^16^ found an association between maternal obesity and an increased risk of a composite outcome of SMM and/or mortality through delivery hospitalization in a cohort of participants who delivered between 2004 through 2013 in Washington State.^16^ Although the results of the present study are similar, the overall incidence of SMM and/or mortality in this study was higher. This difference was not completely explained by the variation in duration of outcome assessment after delivery given that the incidence rates remained higher even when we restricted the duration of follow-up to the delivery hospitalization. Rather, the differences observed may be attributable to the variation in the definition of SMM or the population studied.

Few studies have evaluated obesity and SMM beyond the delivery hospitalization, but such evaluation is critical because a large proportion of pregnancy-related complications occurs after discharge. Between 1% and 2% of postpartum patients in the US are readmitted to the hospital,^25,38,39^ and 14% to 17% of them experience SMM.^25,39^ In addition, more than a third of all pregnancy-related deaths in the US occur between 7 days and 1 year post partum.^23,24^ In a French population-based study that evaluated SMM up to 42 days post partum, investigators found that maternal obesity was associated with antepartum SMM but not with a composite outcome of intrapartum or postpartum SMM.^20^ However, only 12% of participants in the study had a prepregnancy BMI of 30 or higher. Furthermore, postpartum SMM was grouped with intrapartum SMM in the analysis, thus preventing the assessment of SMM risk after delivery.^20^

Despite the importance of maternal mortality as a clinical outcome, it is challenging to study because of the relatively low incidence within the population. In a descriptive study of pregnancy-related deaths in California from 2002 to 2005, individuals who died were more likely to have obesity than the control group (30% vs 16%), but the study did not control for confounders.^17^ Among maternal deaths in New York City between 1995 to 2003 (n = 132), maternal obesity (defined as prepregnancy weight >114 kg or 250 lb) was independently associated with mortality (adjusted odds ratio, 2.9; 95% CI, 1.1-8.1).^12^ However, that study assessed only deaths during the delivery hospitalization.

There were 249 deaths in the present study cohort. Use of data from the Ohio PAMR program to validate maternal death was a major strength of this study given the concerns regarding the accuracy of pregnancy-associated mortality data that rely on vital statistics alone.^40^ The data also allowed us to examine deaths through the first year after delivery and to describe proximate and distal factors associated with mortality within the study cohort. The study was underpowered to evaluate the independent association between BMI and mortality despite the large sample size. Nevertheless, we believe the descriptive data add value to the existing literature.

The mediation analysis highlights the importance of hypertensive disorders in pregnancy among individuals with obesity. We found that 65.1% of the association of maternal obesity with SMM and/or mortality was mediated by hypertension. Leonard et al^14^ similarly reported that maternal comorbidities, including hypertensive conditions, diabetes, and asthma, accounted for a high proportion of SMM in participants with obesity, although the investigators did not evaluate hypertension as an independent factor. Hypertensive diseases in pregnancy complicate 20% of pregnancies among individuals with obesity.^5,41^ It has been estimated that hypertensive diseases are associated with 38% of SMM cases^42^ and 10% of pregnancy-related deaths.^23,24^ Interventions must be developed and implemented to reduce the risk of hypertensive complications as rates of obesity in the population continue to increase.

### Limitations

This study has some limitations. First, the cohort included only pregnancies among beneficiaries of Ohio Medicaid. Although approximately 50% of deliveries in Ohio are covered by Medicaid, the results of this study might not be generalizable to other populations. Second, the incidence of the primary outcome may have been underestimated if the beneficiaries had a lapse in their insurance coverage during pregnancy or after delivery. We attempted to address this possibility by performing a sensitivity analysis that limited the follow-up period to the first 42 days after delivery. Third, although previous validation studies have demonstrated that the data we used have adequate reliability and validity, there was likely some misclassification.^43,44^ Specifically, recent research has shown that the identification of SMM was substantially affected by the transition from *ICD-9* to *ICD-10* codes.^45^ Fourth, although use of large statewide administrative databases allowed for the evaluation of outcomes with relatively low prevalence within the population, the study remained underpowered to enable the evaluation of maternal mortality alone. Fifth, the use of mediation analysis comes with many strict assumptions regarding control of confounding in the various associations between exposure, potential mediator, and outcome; thus, residual confounding could have changed the findings.

## Conclusions

In this large population-based study, maternal prepregnancy obesity was associated with an increased risk of SMM and/or mortality through 1 year post partum. The association between obesity and SMM and/or mortality risk appeared to be mediated by hypertensive disorders. Prevention and management of hypertensive disorders in pregnancy may reduce morbidity and mortality in individuals with obesity.

## Notes

Supplement. **eAppendix.** Additional Methods **eTable 1.** Centers for Disease Control and Prevention List of 21 Indicators Used to Define Severe Maternal Morbidity **eTable 2.** Definitions of Covariates Based on ICD-9/10 Diagnostic and Procedure Codes **eFigure.** Directed Acyclic Graph **eTable 3.** Timing and Cause of Death Based by Maternal Prepregnancy Obesity Status **eTable 4.** Unadjusted Incidence of Severe Maternal Morbidity and Mortality Through 42 Days Postpartum per 10,000 Pregnancies by Maternal Prepregnancy Body Mass Index (BMI) **eTable 5.** Unadjusted Incidence of Severe Maternal Morbidity and Mortality Through Delivery Hospitalization per 10,000 Pregnancies by Maternal Prepregnancy Body Mass Index (BMI) **eTable 6.** Severe Maternal Morbidity by Prepregnancy Maternal Obesity Status and Time Period **eTable 7.** Examination of the Mediating Effects of Maternal Hypertension and Pre-gestational Diabetes in the Relationship Between Maternal Obesity and Maternal Morbidity and Mortality
eReferences
