# Supplementary material for: Association of Prepregnancy Body Mass Index With Risk of Severe Maternal Morbidity and Mortality Among Medicaid Beneficiaries
Source: JAMA Netw Open. 2022 Jun 28;5(6):e2218986. doi: 10.1001/jamanetworkopen.2022.18986 (PMC9240907; doi:10.1001/jamanetworkopen.2022.18986)
Supplement: Supplement. — eAppendix. Additional Methods eTable 1. Centers for Disease Control and Prevention List of 21 Indicators Used to Define Severe Maternal Morbidity eTable 2. Definitions of Covariates Based on ICD-9/10 Diagnostic and Procedure Codes eFigure. Directed Acyclic Graph eTable 3. Timing and Cause of Death Based by Maternal Prepregnancy Obesity Status eTable 4. Unadjusted Incidence of Severe Maternal Morbidity and Mortality Through 42 Days Postpartum per 10,000 Pregnancies by Maternal Prepregnancy Body Mass Index (BMI) eTable 5. Unadjusted Incidence of Severe Maternal Morbidity and Mortality Through Delivery Hospitalization per 10,000 Pregnancies by Maternal Prepregnancy Body Mass Index (BMI) eTable 6. Severe Maternal Morbidity by Prepregnancy Maternal Obesity Status and Time Period eTable 7. Examination of the Mediating Effects of Maternal Hypertension and Pre-gestational Diabetes in the Relationship Between Maternal Obesity and Maternal Morbidity and Mortality eReferences [file jamanetwopen-e2218986-s001.pdf]

Supplemental Online Content

Frey HA, Ashmead R, Farmer A, et al. Association of prepregnancy body mass index with risk of severe maternal morbidity and mortality among Medicaid beneficiaries. *JAMA Netw Open*. 2022;5(6):e2218986. doi:10.1001/jamanetworkopen.2022.18986

eAppendix. Additional Methods

eTable 1. Centers for Disease Control and Prevention List of 21 Indicators Used to Define Severe Maternal Morbidity

eTable 2. Definitions of Covariates Based on ICD-9/10 Diagnostic and Procedure Codes

eFigure. Directed Acyclic Graph

eTable 3. Timing and Cause of Death Based by Maternal Prepregnancy Obesity Status

eTable 4. Unadjusted Incidence of Severe Maternal Morbidity and Mortality Through 42 Days Postpartum per 10,000 Pregnancies by Maternal Prepregnancy Body Mass Index (BMI)

eTable 5. Unadjusted Incidence of Severe Maternal Morbidity and Mortality Through Delivery Hospitalization per 10,000 Pregnancies by Maternal Prepregnancy Body Mass Index (BMI)

eTable 6. Severe Maternal Morbidity by Prepregnancy Maternal Obesity Status and Time Period

eTable 7. Examination of the Mediating Effects of Maternal Hypertension and Pre-gestational Diabetes in the Relationship Between Maternal Obesity and Maternal Morbidity and Mortality

eReferences

This supplemental material has been provided by the authors to give readers additional information about their work.

© 2022 Frey HA et al. *JAMA Network Open.*

eAppendix. Additional Methods

Data Linkage:

We used an extant database of live births (2012-2017) and fetal deaths (2012-2015) linked to Medicaid claims data. To complete the study cohort, we linked 2016 and 2017 fetal deaths to the Medicaid claims database using the same methodology (eAppendix in the Supplement). The probabilistic linkages utilized name, race, date of birth, county, zip code, and address and phone number. After linkage scores were calculated, a manual review was completed to inspect potentially linked pairs. Medicaid claims data were used to further verify linkage results using provider National Provider Identifier (NPI) number and stillbirth diagnosis codes. For maternal deaths, detailed information was obtained from the Ohio Pregnancy-Associated Mortality Review program files. These data were probabilistically linked to the pregnancies in the Medicaid cohort using maternal name, maternal date of birth, birth or fetal death date, and certificate number. All data linkages were carried out with Link Plus software (Link Plus Beta 3.0, Centers for Disease Control and Prevention (CDC), Atlanta, Georgia). The unit of analysis was the pregnancy.

Mediation Analysis:

Maternal hypertensive disease and pre-gestational diabetes were evaluated as potential mediators in the relation between maternal obesity and severe maternal morbidity (SMM) and/or mortality from 20 weeks gestation through one year postpartum using regression-based causal mediation methods.[2] For both hypertension and pre-gestational diabetes, we fit separate mediation models where the relation between the mediator and the covariates was modeled as a logistic regression model and the relation between the outcome and the covariates was modeled as a Poisson regression model.[3] The cohort data were subset to only the last recorded pregnancy per woman (n= 276,691) to fit the mediation models as approaches to account for correlation between observations are not readily accessible. Effect estimates were conditional on specific covariate values and were estimated using the most common pattern of covariates in the data. The multiply imputed covariate data were also used for this analysis.

© 2022 Frey HA et al. *JAMA Network Open.*

eTable 1. Centers for Disease Control and Prevention List of 21 Indicators Used to Define Severe Maternal Morbidity

**CDC – 21 Indicators of Severe Maternal Morbidity[1]**
1. Acute myocardial infarction
2. Aneurysm
3. Acute renal failure
4. Adult respiratory distress syndrome
5. Amniotic fluid embolism
6. Cardiac arrest/ventricular fibrillation
7. Conversion of cardiac rhythm
8. Disseminated intravascular coagulation
9. Eclampsia
10. Heart failure/arrest during surgery or procedure
11. Puerperal cerebrovascular disorders
12. Pulmonary edema/acute heart failure
13. Severe anesthesia complications
14. Sepsis
15. Shock
16. Sickle cell disease with crisis
17. Air and thrombotic embolism
18. Blood product transfusion
19. Hysterectomy
20. Temporary tracheostomy
21. Ventilation

© 2022 Frey HA et al. *JAMA Network Open.*

eTable 2. Definitions of Covariates Based on ICD-9/10 Diagnostic and Procedure Codes

Variable	ICD-9 Codes	ICD-10 Codes
Pre-gestational diabetes	249, 250, 357.2, 362.0, 648.0	E08-E11, E13, O24.01, O24.02, O24.11, O24.12, O24.31, O24.32, O24.81, O24.91
Gestational diabetes	648.8	O24.41, O24.42, O24.43, O99.810
Chronic hypertension	401, 402, 403, 404, 405, 642.0, 642.1, 642.2, 642.7	I10-16, I12.9, O10, O11, O16.1
Pregnancy-associated hypertension (gestational hypertension, preeclampsia or eclampsia)	642.4, 642.5, 642.6, 642.7	O11, O13, O14, O15
Maternal cardiac disease	393-398, 402, 404, 410-416, 420-429, 440, 441, 648.5, 648.6, 745, 746, 747.0, 747.1, 747.2, 747.3, 747.4	I05-09, I25, I27, I28, I30-52, I70, I71, Q20-26, O10.11, O10.31, O99.41X
Illicit substance use	304.X, 305.X 305.1-305.9 (excludes 304.X3 and 305.X3), 648.3X, 292.xx	F11.X, F12.X, F13.X, F14.X, F15.X, F16.X, F18.X, F19.X (excludes F1X.11), O99.32
Chorioamnionitis	658.4	O41.1
Uterine rupture	665.0, 665.1	O71, S37.69XA

© 2022 Frey HA et al. *JAMA Network Open.*

eFigure. Directed Acyclic Graph

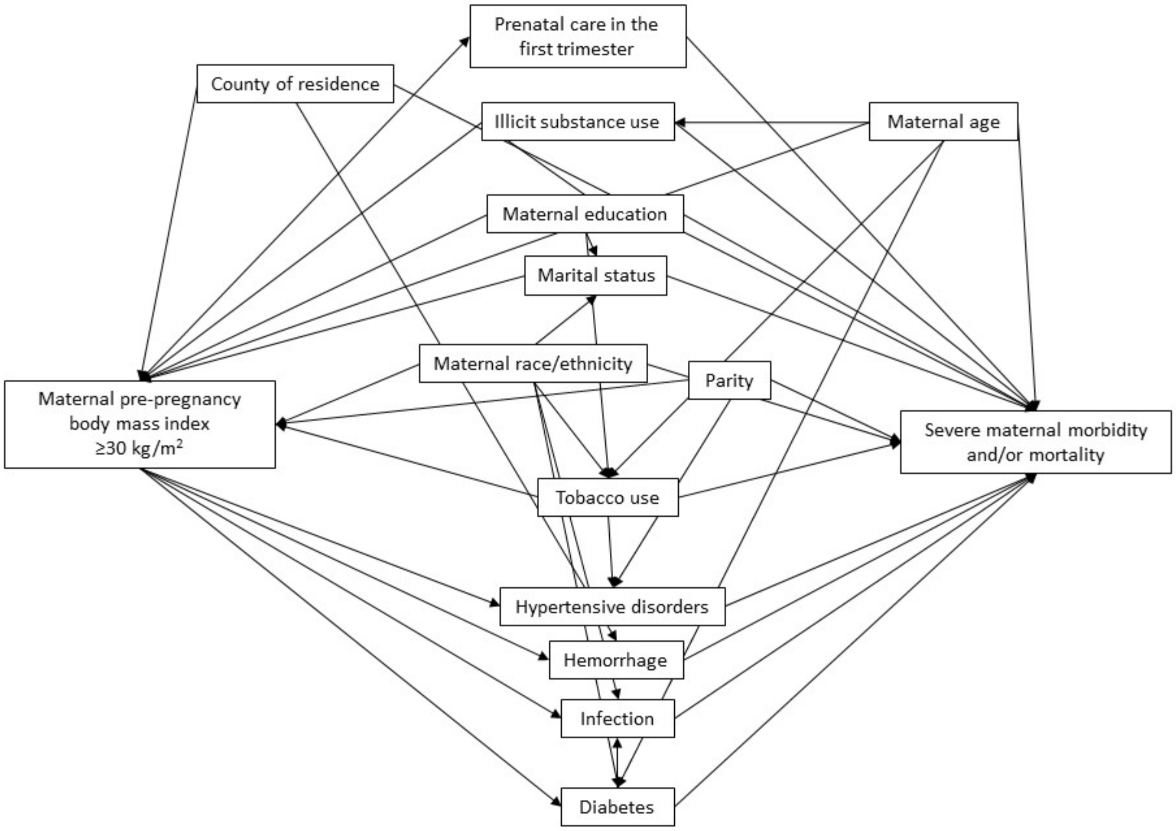

© 2022 Frey HA et al. *JAMA Network Open.*

eTable 3. Timing and Cause of Death Based by Maternal Prepregnancy Obesity Status

	**BMI <30 kg/m^2 (n=170) % (n)**	**BMI ≥30 kg/m^2 (n=79) % (n)**
Classification		
Pregnancy-related	19.4% (33)	30.4% (24)
Pregnancy-associated	64.7% (110)	60.0% (45)
Unable to determine	15.8% (27)	12.7% (10)
Timing		
Antepartum	1.8% (3)	1.2% (1)
Delivery hospitalization	7.6% (13)	5.1% (4)
Postpartum to 42 days	15.3% (26)	17.7% (14)
42 days to 1 year	75.3% (128)	75.9% (60)
Causes of Death	n	n
Amniotic fluid embolism	0	1
Cardiovascular	9	12
Infection	12	3
Hemorrhage	3	2
Hypertension/Eclampsia	4	2
Embolism	2	6
Malignancy	7	4
Homicide	17	5
Suicide	6	4
Unintentional injury	96	33
Other	7	3
Unknown	7	4

© 2022 Frey HA et al. *JAMA Network Open.*

eTable 4. Unadjusted Incidence of Severe Maternal Morbidity and Mortality Through 42 Days Postpartum per 10,000 Pregnancies by Maternal Prepregnancy Body Mass Index (BMI)

Outcome	BMI <18.5 kg/m2 (n=17,476) Rate (n)	BMI 18.5-24.9 kg/m2 (n=140,132) Rate (n)	BMI 25.0-29.9 kg/m2 (n=83,858) Rate (n)	BMI 30.0-34.9 kg/m2 (n=52,245) Rate (n)	BMI 35.0-39.9 kg/m2 (n=28,813) Rate (n)	BMI ≥40 kg/m2 (n=24,973) Rate (n)
Composite of SMM and/or death	343.9 (601)	336.3 (4713)	366.9 (3077)	407.7 (2130)	476.5 (1373)	614.7 (1535)
Composite excluding transfusion	256.4 (448)	258.0 (3616)	296.2 (2484)	343.4 (1794)	406.8 (1172)	549.8 (1373)
Mortality	1.1 (2)	1.6 (23)	2.0 (17)	1.7 (9)	1.4 (4)	2.4 (6)
Acute MI[a]	5.1 (9)	4.1 (58)	4.4 (37)	5.2 (27)	5.9 (17)	7.6 (19)
Aneurysm	2.3 (4)	1.9 (27)	1.7 (14)	3.1 (16)	1.7 (5)	3.2 (8)
Acute renal failure	22.9 (40)	20.3 (284)	24.4 (205)	30.1 (157)	33.0 (95)	38.8 (97)
ARDS[b]	34.3 (60)	33.4 (468)	31.0 (260)	37.1 (194)	47.9 (138)	62.5 (156)
Amniotic fluid embolism	1.1 (2)	1.1 (16)	2.0 (17)	3.1 (16)	1.7 (5)	1.2 (3)
Cardiac arrest/ventricular fibrillation	4.0 (7)	4.4 (61)	4.5 (38)	4.2 (22)	5.2 (15)	5.6 (14)
Conversion of cardiac rhythm	1.7 (3)	1.2 (17)	1.8 (15)	2.5 (13)	1.4 (4)	1.6 (4)
DIC[c]	52.6 (92)	57.2 (801)	58.0 (486)	59.5 (311)	71.5 (206)	76.9 (192)
Eclampsia	33.8 (59)	30.2 (423)	40.1 (336)	51.1 (267)	59.7 (172)	75.3 (188)
Heart failure/arrest in surgery or procedure	4.0 (7)	4.4 (61)	4.5 (38)	4.2 (22)	5.2 (15)	5.6 (14)
Cerebrovascular event	26.9 (47)	26.3 (369)	31.7 (266)	32.2 (169)	36.4 (105)	45.6 (114)
Pulmonary edema/acute heart failure	29.2 (51)	27.8 (390)	36.0 (302)	44.0 (230)	59.3 (171)	91.3 (228)
Severe anesthesia complication	1.1 (2)	1.9 (27)	2.1 (18)	2.9 (15)	1.7 (5)	5.2 (13)
Sepsis	60.1 (105)	62.1 (870)	64.3 (539)	79.4 (415)	97.5 (281)	156.2 (390)
Shock	6.3 (11)	3.8 (53)	4.7 (39)	3.1 (16)	2.8 (8)	2.4 (6)
Sickle cell disease with crisis	20.0 (35)	13.8 (194)	11.4 (96)	14.4 (75)	14.6 (42)	18.4 (46)
Air and thrombotic embolism	20.6 (36)	22.5 (315)	34.8 (292)	37.1 (194)	50.7 (146)	77.7 (194)
Blood transfusion	109.3 (191)	98.5 (1380)	89.7 (752)	84.8 (443)	91.6 (264)	89.7 (224)
Hysterectomy	9.7 (17)	8.1 (114)	9.3 (78)	12.4 (65)	13.9 (40)	15.2 (38)
Temporary tracheostomy	2.3 (4)	0.4 (6)	0.5 (4)	0.2 (1)	0.7 (2)	0.4 (1)
Ventilation	25.2 (44)	16.3 (228)	14.7 (123)	16.7 (87)	22.6 (65)	33.2 (83)

© 2022 Frey HA et al. *JAMA Network Open.*

[a]MI denotes myocardial infarction

[b]ARDS denotes acute respiratory distress syndrome

[c]DIC denotes disseminated intravascular coagulation.

© 2022 Frey HA et al. *JAMA Network Open.*

eTable 5. Unadjusted Incidence of Severe Maternal Morbidity and Mortality Through Delivery Hospitalization per 10,000 Pregnancies by Maternal Prepregnancy Body Mass Index (BMI)

Outcome	BMI <18.5 kg/m2 (n=17,476) Rate (n)	BMI 18.5-24.9 kg/m2 (n=140,132) Rate (n)	BMI 25.0-29.9 kg/m2 (n=83,858) Rate (n)	BMI 30.0-34.9 kg/m2 (n=52,245) Rate (n)	BMI 35.0-39.9 kg/m2 (n=28,813) Rate (n)	BMI ≥40 kg/m2 (n=24,973) Rate (n)
Composite of SMM and/or death	266.7 (466)	252.7 (3541)	265.0 (2222)	274.0 (1435)	326.9 (942)	378.4 (945)
Composite excluding transfusion	183.7 (321)	174.7 (2448)	193.1 (1619)	209.6 (1095)	257.9 (743)	313.5 (783)
Mortality	0.6 (1)	0.7 (10)	0.6 (5)	0.6 (3)	0	0.8 (2)
Acute MI[a]	2.9 (5)	2.3 (32)	2.5 (21)	3.6 (19)	4.2 (12)	5.2 (13)
Aneurysm	1.7 (3)	1.8 (25)	1.4 (12)	2.5 (13)	1.7 (5)	2.0 (5)
Acute renal failure	16.0 (28)	14.0 (196)	16.9 (142)	20.5 (107)	21.9 (63)	24.4 (61)
ARDS[b]	25.7 (45)	23.9 (335)	20.9 (175)	25.6 (134)	33.3 (96)	39.2 (98)
Amniotic fluid embolism	1.1 (2)	1.1 (15)	2.0 (17)	2.9 (15)	1.4 (4)	1.2 (3)
Cardiac arrest/ventricular fibrillation	1.7 (3)	2.4 (33)	2.6 (22)	3.3 (17)	3.8 (11)	2.8 (7)
Conversion of cardiac rhythm	0.6 (1)	1.0 (14)	1.7 (14)	2.3 (12)	1.4 (4)	1.6 (4)
DIC[c]	46.3 (81)	49.7 (697)	50.3 (422)	53.0 (277)	63.9 (184)	62.5 (156)
Eclampsia	22.9 (40)	18.3 (257)	24.2 (203)	28.1 (147)	36.8 (106)	45.6 (114)
Heart failure/arrest in surgery or procedure	1.7 (3)	2.4 (33)	2.6 (22)	3.3 (17)	3.8 (11)	2.8 (7)
Cerebrovascular event	17.7 (31)	16.8 (236)	21.3 (179)	18.2 (95)	25.0 (72)	29.6 (74)
Pulmonary edema/acute heart failure	20.6 (36)	16.8 (236)	19.1 (160)	21.2 (111)	34.4 (99)	44.0 (110)
Severe anesthesia complication	1.1 (2)	1.4 (20)	1.8 (15)	1.7 (9)	0.7 (2)	3.6 (9)
Sepsis	26.3 (46)	23.3. (327)	20.9 (175)	22.0 (115)	25.7 (74)	27.6 (69)
Shock	5.7 (10)	3.7 (52)	4.2 (35)	2.9 (15)	2.8 (8)	2.0 (5)
Sickle cell disease with crisis	14.3 (25)	10.1 (142)	7.4 (62)	10.7 (56)	11.5 (33)	13.6 (34)
Air and thrombotic embolism	9.7 (17)	14.6 (205)	21.5 (180)	22.0 (115)	30.9 (89)	48.5 (121)
Blood transfusion	97.8 (171)	93.5 (1310)	85.6 (718)	80.4 (420)	85.7 (247)	81.3 (203)
Hysterectomy	8.0 (14)	7.1 (99)	8.2 (69)	12.1 (63)	13.5 (39)	14.8 (37)
Temporary tracheostomy	1.1 (2)	0.1 (2)	0.2 (2)	0.2 (1)	0.3 (1)	0
Ventilation	20.0 (35)	13.1 (183)	12.5 (105)	14.2 (74)	19.4 (56)	26.4 (66)

[a]MI denotes myocardial infarction

[b]ARDS denotes acute respiratory distress syndrome

[c]DIC denotes disseminated intravascular coagulation.

© 2022 Frey HA et al. *JAMA Network Open.*

eTable 6. Severe Maternal Morbidity by Prepregnancy Maternal Obesity Status and Time Period[a]

	Antepartum<br><br>n (%)	Delivery hospitalization<br>n (%)	Discharge to 42 days<br>n (%)	42 days to one year<br>n (%)
Severe maternal morbidity				
BMI <30 kg/m^2	2287 (0.9%)	4295 (1.8%)	2648 (1.1%)	4178 (1.7%)
BMI ≥30 kg/m^2	1389 (1.3%)	2187 (2.1%)	2087 (2.0%)	2524 (2.4%)
Severe maternal morbidity without transfusion				
BMI <30 kg/m^2	2217 (0.9%)	2485 (1.0%)	2596 (1.1%)	4065 (1.7%)
BMI ≥30 kg/m^2	1365 (1.3%)	1493 (1.4%)	2067 (1.9%)	2485 (2.3%)

[a]Note: time periods are not mutually exclusive. Individuals can contribute data in more than one time period.

© 2022 Frey HA et al. *JAMA Network Open.*

eTable 7. Examination of the Mediating Effects of Maternal Hypertension and Pre-gestational Diabetes in the Relationship Between Maternal Obesity and Maternal Morbidity and Mortality

	**Hypertensive disorders**				**Pre-gestational diabetes**			
	aRR TE[a,b] (95% CI)	aRR NDE[a,b] (95% CI)	aRR NIE[a,b] (95% CI)	Percent Mediated (95% CI)	aRR TE[a,b] (95% CI)	aRR NDE[a,b] (95% CI)	aRR NIE[a,b] (95% CI)	Percent Mediated (95% CI)
SMM and/or mortality	1.35 (1.30, 1.40)	1.12 (1.08, 1.16)	1.20 (1.19, 1.22)	65.1% (64.6%, 65.6%)	1.32 (1.28, 1.37)	1.29 (1.25, 1.34)	1.02 (1.02, 1.03)	9.1% (8.7%, 9.4%)
SMM and/or mortality (without transfusion)	1.43 (1.38, 1.48)	1.18 (1.14, 1.22)	1.21 (1.20, 1.23)	58.5% (58.0%, 59.0%)	1.40 (1.35, 1.45)	1.37 (1.32, 1.42)	1.02 (1.02, 1.03)	8.0% (7.7%, 8.4%)

[a]Adjusted for maternal age, race/ethnicity, education level, marital status, parity, tobacco use, illicit substance use, prenatal care in the first trimester and county of residence.

[b]95% CI denotes 95% confidence interval. TE denotes total effect. NDE denotes natural direct effect and NIE denotes natural indirect effect.

© 2022 Frey HA et al. *JAMA Network Open.*

eReferences

1. Centers for Disease Control and Prevention. Severe Maternal Morbidity in the United States. https://www.cdc.gov/reproductivehealth/maternalinfanthealth/severematernalmorbidity.html. Accessed 6/3/2020.

2. Valeri L, Vanderweele TJ. Mediation analysis allowing for exposure-mediator interactions and causal interpretation: theoretical assumptions and implementation with SAS and SPSS macros. *Psychol Methods* 2013;18(2):137-150.

3. Conducting regression-based causal medication analysis using the R package "regmedint". OSF Preprints; 2020.

© 2022 Frey HA et al. *JAMA Network Open.*
